# Unmasking

**DOI:** 10.1038/s41390-023-02815-8

**Published:** 2023-09-12

**Authors:** Michele Kong, Chelsea Brown

**Affiliations:** 1 The University of Alabama at Birmingham, Birmingham, AL USA; 2 Children’s of Alabama, Birmingham, AL USA

In a recent organization-wide email, our hospital’s leadership shared the institution’s next big step toward a “return to normal” outlining the discontinuation of a 3-year universal masking mandate. Similarly, throughout the country, almost all medical facilities are lifting their masking requirements. Though masks have served an important role during the COVID-19 pandemic, many providers have shared feelings of excitement that unmasking will allow them to finally feel fully seen by their patients, families, and co-workers.

While universal masking may now become a pandemic memory, the concept of “masking” and “unmasking” is not new to the neurodivergent community. Masking, also known as camouflaging, is considered a social practice whereby individuals hide certain traits or behaviors of their true selves that may be considered non-neurotypical to meet social expectations and to avoid stigma. This may take the form of suppressing behaviors such as “stimming” that helps with sensory regulation, internalizing different feelings or moods, scripting conversations for interactions with others, and more.

In the summer of 2020, I found myself faced with the fallout of having a stroke and hemifacial spasm. The mask, a simple item, meant to protect me and my patients from COVID-19 became part of my defense mechanism, my refuge, allowing me to hide the asymmetry in my face, the facial droop and constant twitching. And now, without the physical mask, I find myself creating an invisible one, in an attempt to hide my disability. Some days, a part of me feels especially burdened, and unwilling to let go of my own self-judgment. Not long ago during an interview, I was asked if I had a favorite restaurant. Although I could picture the restaurant as clear as day, I struggled to retrieve the name, and finally gave a generic response to fill the awkward pause. Photos and videos taken to capture memories instead become harsh reminders of how I was before the diagnosis. Stares from strangers or comments from well-intending friends about the spasms compound the feeling of self-consciousness and embarrassment about my inability to control these contractions. Recently, a friend shared a viral video clip of Lewis Capaldi, a singer who had Tourette-induced tics that prohibited him from singing. I watched with tears streaming down my face as the public helped him finish his song (https://www.foxnews.com/entertainment/lewis-capaldis-fans-finish-song-singer-suffers-tourettes-episode-concert-germany). The burden of having a disability, visible or not, is real and can impact anyone across the age spectrum, diagnosis, socioeconomic status, gender, or nationality. This burden is further magnified when individuals feel isolated, or when their neurodivergent behavior is stigmatized. We need to not only increase our society’s awareness about these atypical movements or behaviors, but also to examine the negative effects that masking has on neurodivergent individuals and their families. In a self-reported study, adults with autism commonly report huge emotional and physical tolls related to masking including feelings of burnout, identity, and relational issues, and increased suicidality.^1^

In the healthcare setting, for our patients with autism and other sensory processing challenges, attempting to mask or redirect behaviors that help with coping can magnify feelings of stress and anxiety which can negatively influence medical assessment and treatment. Furthermore, these patients often have additional barriers related to communication, comprehension, and an insistence on sameness and routine, all of which may be heightened when they are feeling unwell in an environment that is foreign and unpredictable. In addition, caregivers of neurodivergent children may experience their own feelings of stress and anxiety revolving around societal reactions to their child’s responses and behaviors.^2^ Whether consciously or not, they may come to the healthcare setting anticipating a difficult visit. These feelings are often reinforced by experiences that were previously negative or unwelcoming in nature. When paired with challenging non-accommodating encounters, these factors may create barriers to appropriate care.^3^

As healthcare providers, we need to alleviate the pressure that our patients feel in trying to mask their differences, especially during a period of illness when stress and trepidation are likely to be high. By promoting awareness of sensory-specific needs of our patients, and providing training opportunities to medical staff, we equip them with the knowledge and skillset necessary to appropriately triage, treat and manage patients who might be experiencing sensory overload while seeking medical help for their acute illness. For instance, a child who is easily overwhelmed by loud noises might demonstrate proprioceptive defensiveness by cupping their ears and bouncing repeatedly on the exam table. Instead of asking the child to be quiet or to stop bouncing, the physician might instead offer noise-canceling headphones, and incorporate the patient’s “bounciness” into their physical exam routine.

By learning about and making available sensory resources (for instance, headphones, fidget tools or mobile sensory stations) that may help their patients, the medical staff can position themselves to be aware of baseline behaviors and needs of their patients, and therefore become better equipped to create an environment where those needs and behaviors are accepted and embraced. When patients feel empowered to unmask and share their true selves, we are gifted with the opportunity to develop focused care plans through a strengths-based lens. As practitioners, our goal is not just to treat the illness, but to treat the child who has the illness. This means understanding what makes the child unique and using that information to create a care plan and environment that is individualized to them.

As a community, we need to open our minds and our hearts. Unless we start embracing everyone for who they are, whether they conform to what we perceive as societal norms or not, we will never have true inclusion. We must create an environment that is judgment free, that allows for everyone to be the best versions of themselves. Such an environment will allow those of us with disabilities to embrace our own limitations, be vulnerable, be ready to forgive ourselves, and to let go of any self-judgments.

A family member said it best during a recent visit to our hospital. “Instead of assuming that our son must respond as a neurotypical child, the staff chose to look deeper. They saw that he loved to push, so they gave him the role of pushing his bed to surgery. They saw that his love for opening and closing doors was a soothing behavior and not a misbehavior. The staff was prepared in advance to better understand his needs, and it made all the difference.”

Let us all be our authentic selves and leave the mask behind once and for all.
